# Molecular Mechanisms of Adiponectin-Induced Attenuation of Mechanical Stretch-Mediated Vascular Remodeling

**DOI:** 10.1155/2020/6425782

**Published:** 2020-05-21

**Authors:** Crystal M. Ghantous, Rima Farhat, Laiche Djouhri, Sarah Alashmar, Gulsen Anlar, Hesham M. Korashy, Abdelali Agouni, Asad Zeidan

**Affiliations:** ^1^Department of Anatomy, Cell Biology and Physiology, Faculty of Medicine, American University of Beirut, Beirut, Lebanon; ^2^Department of Nursing and Health Sciences, Faculty of Nursing and Health Sciences, Notre Dame University-Louaize, Beirut, Lebanon; ^3^Department of Basic Sciences, College of Medicine QU Health, Qatar University, Doha, Qatar; ^4^Department of Pharmaceutical Sciences, College of Pharmacy, QU Health, Qatar University, Doha, Qatar

## Abstract

Hypertension induces vascular hypertrophy, which changes blood vessels structurally and functionally, leading to reduced tissue perfusion and further hypertension. It is also associated with dysregulated levels of the circulating adipokines leptin and adiponectin (APN). Leptin is an obesity-associated hormone that promotes vascular smooth muscle cell (VSMC) hypertrophy. APN is a cardioprotective hormone that has been shown to attenuate hypertrophic cardiomyopathy. In this study, we investigated the molecular mechanisms of hypertension-induced VSMC remodeling and the involvement of leptin and APN in this process. To mimic hypertension, the rat portal vein (RPV) was mechanically stretched, and the protective effects of APN on mechanical stretch-induced vascular remodeling and the molecular mechanisms involved were examined by using 10 *μ*g/ml APN. Mechanically stretching the RPV significantly decreased APN protein expression after 24 hours and APN mRNA expression in a time-dependent manner in VSMCs. The mRNA expression of the APN receptors AdipoR1, AdipoR2, and T-cadherin significantly increased after 15 hours of stretch. The ratio of APN/leptin expression in VSMCs significantly decreased after 24 hours of mechanical stretch. Stretching the RPV for 3 days increased the weight and [^3^H]-leucine incorporation significantly, whereas APN significantly reduced hypertrophy in mechanically stretched vessels. Stretching the RPV for 10 minutes significantly decreased phosphorylation of LKB1, AMPK, and eNOS, while APN significantly increased p-LKB1, p-AMPK, and p-eNOS in stretched vessels. Mechanical stretch significantly increased p-ERK1/2 after 10 minutes, whereas APN significantly reduced stretch-induced ERK1/2 phosphorylation. Stretching the RPV also significantly increased ROS generation after 1 hour, whereas APN significantly decreased mechanical stretch-induced ROS production. Exogenous leptin (3.1 nM) markedly increased GATA-4 nuclear translocation in VSMCs, whereas APN significantly attenuated leptin-induced GATA-4 nuclear translocation. Our results decipher molecular mechanisms of APN-induced attenuation of mechanical stretch-mediated vascular hypertrophy, with the promising potential of ultimately translating this protective hormone into the clinic.

## 1. Introduction

Being a disease itself, hypertension is also a major risk factor for the development of other cardiovascular diseases, such as stroke, renal disease, and heart failure [1, 2]. In response to hypertension, small resistance vessels undergo vascular hypertrophy and remodeling [3]; their walls become thicker, stiffer, and less elastic, increasing the risk of vascular blockage and rupture, and potentially leading to organ damage and failure [4, 5]. Hypertension is not only associated with cardiovascular abnormalities in structure and function but also with dysregulated circulating levels of two important adipokines: leptin and adiponectin (APN). Plasma leptin levels are increased while APN levels are decreased in hypertensive patients [6–8]. After their discovery and for a while, these adipokines were believed to be almost exclusively produced by adipocytes [9–15], but studies later showed that they are also produced by other kinds of cells, including cardiomyocytes [16–18]. However, little research has been done on whether leptin and APN are also produced by VSMCs and whether their expression is affected by hypertension.

Leptin is a hormone whose levels are directly associated with obesity [19], myocardial infarction [20], and hypertension [7, 21]. Studies have also shown a direct involvement of leptin in promoting hypertension-induced vascular hypertrophy [22, 23]. Leptin is synthesized by VSMCs in response to forces that mimic hypertension and, in turn, induces VSMC hypertrophy [22–24]. This hormone exerts a prohypertrophic effect on VSMCs by activating several signaling pathways and transducers, including the MAPK ERK1/2 [23], the RhoA/ROCK pathway [22, 25], and the prohypertrophic transcriptional factors serum response factor (SRF) and GATA-4 [22, 25, 26]. In addition, leptin-induced vascular remodeling has been associated with increased reactive oxygen species (ROS) production in the vascular wall [22]. Hypertension increases ROS production in VSMCs [27, 28], and this is partly mediated by leptin [22]. In turn, ROS induce hypertrophy of VSMCs [29, 30].

As opposed to leptin, APN levels are inversely associated with obesity [31–33], myocardial infarction [34, 35], and hypertension [8, 36]. Moreover, APN supplementation has been shown to be cardioprotective; for instance, APN administration protects against myocardial injury after ischemia-reperfusion and inhibits pressure overload-induced cardiac hypertrophy [37, 38]. To elicit its intracellular effects, APN can bind to three receptors: APN receptor 1 (AdipoR1), APN receptor 2 (AdipoR2), and T-cadherin [39–41]. Knockout mice lacking AdipoR1 and R2 have increased oxidative stress, inflammation, triglyceride content, glucose intolerance, and insulin resistance, indicating the predominant role of these receptors in mediating the metabolic effects of APN [42]. T-cadherin-null mice have exaggerated cardiac hypertrophy in response to pressure overload [43] and impaired revascularization in response to ischemia [44].

Binding of APN to its receptors activates 5′-AMP-activated protein kinase (AMPK) signaling, which plays a role in glucose utilization, insulin sensitivity, and fatty-acid oxidation [45]. AMPK is mainly activated by its upstream enzyme liver kinase B1 (LKB1), which is first activated by getting phosphorylated at its Ser428 residue [46, 47]. In turn, LKB1 activates AMPK by phosphorylating it at the Thr172 residue in the activation loop of the *α* subunit [46, 48, 49]. AMPK activation has been shown to exert protective actions, such as attenuating VSMC hypertrophy [50], improving endothelial function [51], and reducing agonist-induced blood pressure [52]. APN also stimulates the production of nitric oxide (NO) in endothelial cells by activating endothelial nitric oxide synthase (eNOS) [53, 54], a process that is mediated by AMPK activation [53]. As a result, more NO is produced to induce VSMC relaxation.

The goal of this research was to investigate the molecular mechanisms of hypertension-induced VSMC remodeling and the involvement of leptin and APN in this process. Moreover, APN's potential protective effect against hypertension-induced vascular remodeling and the mechanisms involved were examined. In order to achieve these aims, the rat portal vein (RPV) was mechanically stretched in a well-characterized organ culture model to mimic hypertension [23, 25, 55–57]. The RPV has distinct musculature; its tunica media is composed of an outer, thick layer of longitudinally oriented VSMCs, whereas its inner, thin layer has circularly oriented VSMCs [58, 59]. In order to mimic hypertension, the RPV was stretched with weights that lead to 10-15% stretch, which has been calculated using the force-length relationship [57, 58, 60]. Moreover, the RPV exhibits spontaneous myogenic tone and contractile activity [57, 58], and accordingly, this vessel has been used as an analogue for small precapillary resistance blood vessels [61]. Since physiological concentrations of APN range between 5 and 25 *μ*g/ml [62], 10 *μ*g/ml of exogenous APN was used to examine the potential protective effect of APN on mechanical stretch-induced VSMC hypertrophy and the molecular mechanisms involved, including LKB1-AMPK signaling, ERK1/2 activation, ROS production, and GATA-4 nuclear translocation.

## 2. Materials and Methods

### 2.1. Rat Portal Vein Organ Culture

Male Sprague-Dawley rats (200-250 gr) were euthanized using CO_2_, as approved by The Animal Care Program and the Institutional Animal Care and Use Committee at the Faculty of Medicine, American University of Beirut. The RPV was dissected out in a sterile environment, placed in an ice-cold N-Hepes buffer solution (400 mM NaCl, 200 mM KCl, 100 mM MgCl_2_, 100 mM Hepes, 11.5 mM Glucose, and 5% penicillin-streptomycin), stripped of its surrounding adipose and connective tissue, and denuded using forceps. It was then cut longitudinally into two halves. To mechanically stretch the RPV, silver weights of 0.6 grams (stretch the RPV slightly above optimal length) were tied to the end of one RPV strip, while the other was left unstretched and used as a negative control. The RPVs were then transferred to culture media of Dulbecco's Modified Eagle's Medium (DMEM)/F-12 HAM with 5% penicillin-streptomycin and incubated at 37°C, 5% CO_2_ in air.

Since physiological concentrations of APN range between 5 and 25 *μ*g/ml [62], 10 *μ*g/ml of exogenous APN (Santa Cruz Biotechnology, California, USA) was used. APN was added to the culture media 1 hour before mechanical stretch was applied or agonists were added. Following incubation, the RPVs were taken out of the incubator and immediately either snap-frozen in liquid nitrogen for protein analysis, weighed, or embedded in frozen blocks and cut cross-sectionally for histological examination.

To measure changes in wet weight, RPVs were weighed before organ culture. They were then blotted gently using a filter paper and weighed after culture, as previously described [57]. To confirm that the changes in weight were due to actual hypertrophy and not just osmosis, dry weight/wet weight ratios were calculated. Dry weight was determined after placing the cultured RPVs at 100°C for 24 hours and then weighing immediately. The dry weight/wet weight ratio was calculated by the ratio of the dry weight value to the wet weight after culture.

### 2.2. Immunoblotting

Protein extraction, sodium dodecyl sulfate polyacrylamide gel electrophoresis (SDS-PAGE), and Western blotting were done as previously described [22, 63]. Primary antibodies for APN (sc-26497), leptin (sc-842), p-LKB1 (cell-3482), p-AMPK (cell-2535), p-ERK1/2 (sc-81492), T-ERK1/2 (sc-292838), p-eNOS (sc-12972), actin (sc-1616), and GAPDH (sc-32233) were supplied by Santa Cruz Biotechnology (California, USA) or Cell Signaling Technology (Massachusetts, USA) and were added at 1 : 500 or 1 : 1000 ratio in 5% BSA for 1 hour.

### 2.3. Immunohistochemistry for APN Expression

To visualize the expression of APN, 5 *μ*m thick cryosections were fixed using freshly prepared 4% paraformaldehyde for 15 minutes, rinsed twice with PBS, and permeabilized using 0.2% Triton X-100 in PBS for 20 minutes. The blocking solution (1% BSA, 0.1% Triton X-100 in PBS) was added for 1 hour to block nonspecific binding. Anti-APN (sc-26497, Santa Cruz Biotechnology, California, USA) primary antibody was then added at 1 : 100 ratio in 1% BSA, 0.05% Tween-20, in PBS and placed overnight at 4°C. The sections were then washed 5 times for 10 minutes each using 0.1% Tween-20 in PBS and then probed for 1 hour in the dark with donkey anti-goat secondary antibody conjugated to CruzFluor 594 (sc-362275, 1 : 250 ratio in 1% BSA, 0.05% Tween-20 in PBS, Santa Cruz Biotechnology, California, USA). Sections were then rinsed 5 times for 10 minutes each with 0.1% Tween-20 in PBS. The mounting media containing the nuclear counterstain 4′,6-diamidino-2-phenylindole (DAPI) (UltraCruz Hard-set Mounting Medium, sc-359850, Santa Cruz Biotechnology, Texas, USA) was then added for 20 minutes in the dark, and images were acquired with a laser scanning confocal microscope (LSM710, Carl Zeiss, Germany). APN positive intensity was quantified using ZEN software (Carl Zeiss, 2012).

### 2.4. RNA Extraction and Real-Time PCR

RNA extraction and real-time PCR analysis were performed as previously described [23]. The used primers were as follows: APN forward 5′-TCCCTCCACCCAAGGAAACT-3′ and APN reverse 5′-TTGCCAGTGCTGCCGTGATA-3′, AdipoR1 forward 5′-GCTGGCCTTTATGCTGCTCG-3′ and AdipoR1 reverse 5′-TCTAGGCCGTAACGGAATTC-3′, AdipoR2 forward 5′-CCACAACCTTGCTTCATCTA-3′ and AdipoR2 reverse 5′-GATACTGAGGGGTGGCAAAC-3′, T-cadherin forward 5′-TCGGGTCTGTCACTATCAAC-3′ and T-cadherin reverse 5′-TGAGGTCTCAAGCCCATAC-3′, and the housekeeping gene 18S rRNA forward 5′-GTAACCCGTTGAACCCCATT-3′ and 18S rRNA reverse 5′-CCATCCAATCGGTAGTAGCG-3′.

### 2.5. Protein Synthesis Measurement

Protein synthesis was measured by assessing [^3^H]-leucine incorporation. RPVs were cultured for 2 days, followed by adding radioactively labelled [^3^H]-leucine (Activity: 1 *μ*Ci/ml; Amersham, Illinois, USA) in the media for an additional day. [^3^H]-leucine incorporation was measured by liquid scintillation counting, as described previously [57].

### 2.6. ROS Analysis

RPV sections (5 *μ*m thickness) were stained with dihydroethidium (DHE) (Invitrogen, Oregon, USA) at a concentration of 10 *μ*M in N-Hepes buffer and incubated at 37°C, 5% CO_2_, for 30 minutes protected from light. The mounting media containing DAPI (UltraCruz Hard-set Mounting Medium, sc-359850, Santa Cruz Biotechnology, Texas, USA) was then added for 20 minutes in the dark. Images were acquired and DHE fluorescence intensity was quantified using a laser scanning confocal microscope (LSM710, Carl Zeiss, Germany) and ZEN software (Carl Zeiss, 2012).

### 2.7. Determination of GATA-4 Nuclear Translocation by Immunofluorescence

Rat aortic smooth muscle cells (RASMCs) were cultured (50 × 10^3^ − 100 × 10^3^ cells/ml) in complete media (DMEM (1 g/l glucose), 10% fetal bovine serum, 1% penicillin-streptomycin, 2 mM Glutamine, 20 mM Hepes) and incubated at 37°C, 5% CO_2_, for 3 days, followed by serum-starvation. The next day, the RASMCs were treated with exogenous APN (10 *μ*g/ml) and leptin (3.1 nM; equivalent to approximately 50 ng/ml). When the treatment was over, the media was aspirated and the cells were fixed with freshly prepared 4% paraformaldehyde for 15 minutes. Permeabilization was performed using 0.2% Triton X-100 for 30 minutes and blocking was done using 1% BSA, 0.1% Triton X-100, in PBS for 1 hour. The primary antibody for GATA-4 (sc-25310, Santa Cruz Biotechnology, California, USA) was added at 1 : 100 ratio in 1% BSA, 0.05% Tween-20, in PBS overnight at 4°C. Washing then followed, where 0.1% Tween-20 in PBS was added 5 times for 10 minutes each. The secondary antibody was CruzFluor 488-conjugated goat anti-mouse antibody (sc-362257, Santa Cruz Biotechnology, California, USA) used at 1 : 250 ratio in 1% BSA, 0.05% Tween-20, in PBS, which was added for 1 hour in the dark. The cells were rinsed again 5 times for 10 minutes each using 0.1% Tween-20 in PBS. To stain actin, phalloidin (100 nM; Acti-stain 555 phalloidin, Cytoskeleton, Colorado, USA) was added for 20 minutes followed by rinsing twice with 0.1% Tween-20 in PBS. Finally, the mounting media containing DAPI (UltraCruz Hard-set Mounting Medium, sc-359850, Santa Cruz Biotechnology, Texas, USA) was added for 20 minutes in the dark. Images were acquired using the Zeiss Axio Observer Z1 microscope (Carl Zeiss, Germany), and data were analyzed using ZEN software (Carl Zeiss, 2012). The purity of the cells as RASMCs was confirmed by immunostaining for alpha-smooth muscle actin (*α*-SMA).

### 2.8. Statistical Analysis

The results are presented as fold change with respect to the negative control, which was unstretched or untreated. The statistical analysis software SigmaStat (Systat Software, California, USA) was used to compute the mean and standard error of the mean (SEM) for each group. To compare 2 groups, *t*-test was used, while one-way analysis of variance (ANOVA) was used to compare 3 or more groups. The data are presented as mean ± SEM for each group in graphs using the graphing software SigmaPlot (Systat Software, California, USA). The difference between groups was considered to be statistically significant if *p* values were less than 0.05 (statistical significance: *p* < 0.05).

## 3. Results

### 3.1. Mechanical Stretch Reduces APN Expression in VSMCs

**Figure 1:**
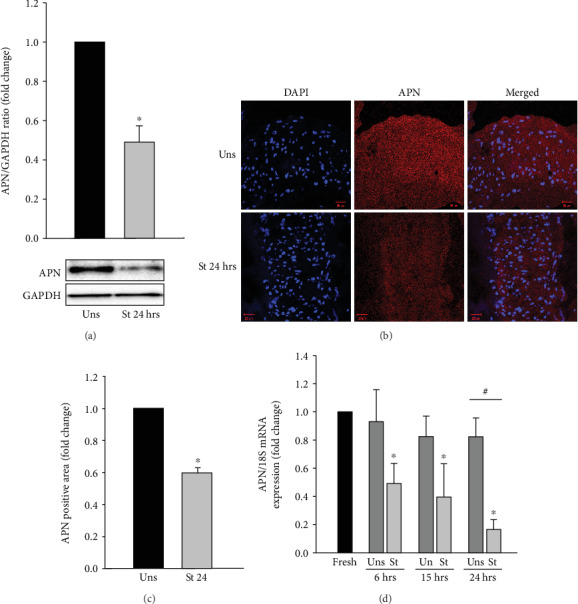
Mechanical stretch-induced downregulation of APN protein and mRNA expression in VSMCs. RPVs were stretched (St) for 24 hours or left unstretched (Uns). (a) APN protein expression was evaluated by Western blot and normalized to the unstretched RPVs. (b) Cryosections of the RPV wall were probed with primary anti-APN antibody and secondary antibody to mark APN (red). DAPI was used to stain the nuclei blue *(40x)*. (c) APN fluorescence intensity was measured using ZEN software and normalized to the unstretched RPVs. ^∗^*p* < 0.05 versus unstretched. (d) Real-time PCR analysis was performed to examine APN mRNA expression in stretched RPVs for 6, 15, or 24 hours as well as unstretched and fresh RPVs. Data were normalized to the fresh RPVs. Results are represented as mean ± SEM. *n* = 4 − 8. ^∗^*p* < 0.05 versus fresh. ^#^*p* < 0.05 versus unstretched.

Hypertension is associated with reduced circulating levels of APN [8], which is mainly known to be produced by adipocytes [11, 15, 64]. To our knowledge, whether VSMCs produce APN and whether hypertension dysregulates its potential production in VSMCs have not been fully elucidated yet. To investigate this, RPVs were either mechanically stretched or left unstretched for 24 hours, followed by Western blot analysis. As shown in Figure 1(a), mechanically stretching the RPV for 24 hours significantly decreased APN expression compared to the control.

The ability of VSMCs to produce APN and the effect of mechanical stretch on APN expression in VSMCs were further examined by immunofluorescence. RPVs were stretched for 24 hours or left unstretched, cut into 5 *μ*m thick cryosections, and probed with anti-APN antibody to mark APN. DAPI was used to stain the nuclei. APN positive intensity was measured using ZEN software (Carl Zeiss, 2012). In agreement with the Western blot findings, APN expression in the mechanically stretched RPVs was significantly reduced compared to the unstretched RPVs (Figures 1(b) and 1(c)).

To examine whether the decrease in intracellular APN levels in response to mechanical stretch occurred at the transcriptional level, real-time PCR analysis was done to examine the effect of stretch on APN mRNA expression in VSMCs. RPVs were mechanically stretched for 6, 15, or 24 hours, and their APN mRNA expression levels were compared to those of unstretched RPVs for 6, 15, or 24 hours and fresh RPVs. As shown in Figure 1(d), mechanical stretch for 6 hours caused a significant decrease in APN mRNA expression compared to fresh RPVs. Stretch for 15 hours caused a more pronounced and significant decrease in APN mRNA expression, while mechanically stretching the RPV for 24 hours led to an even more pronounced reduction in APN mRNA expression compared to fresh and unstretched RPVs for 24 hours (Figure 1(d)).

### 3.2. Mechanical Stretch Increases the APN Receptors' mRNA Expression in VSMCs

**Figure 2:**
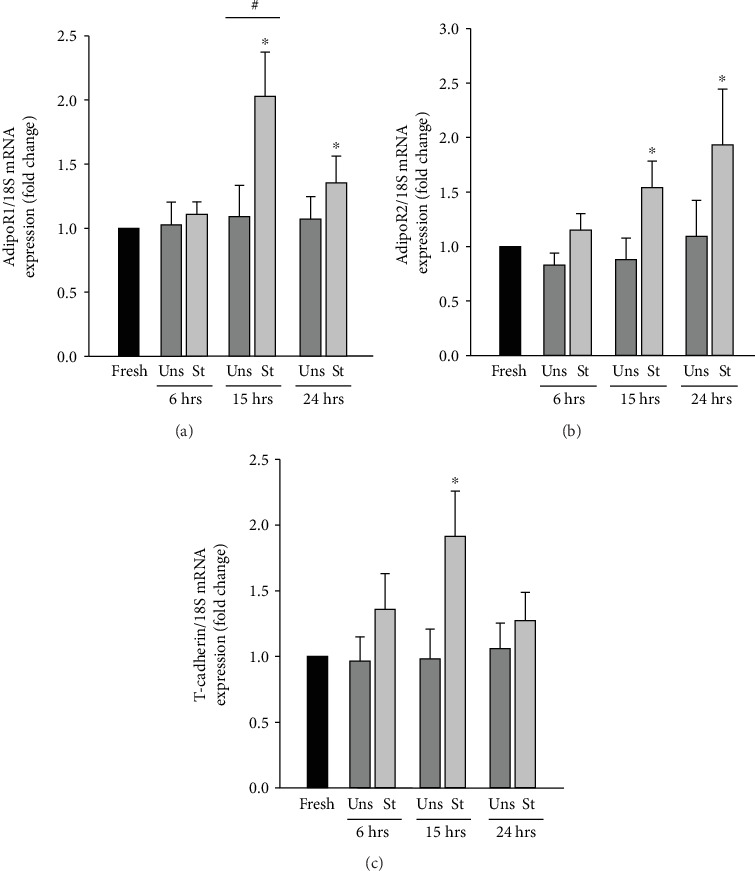
Mechanical stretch-induced increase in the mRNA expression of the APN receptors in VSMCs. RPVs were mechanically stretched (St) for 6, 15, or 24 hours or left unstretched (Uns), followed by real-time PCR analysis to examine AdipoR1 (a), AdipoR2 (b), and T-cadherin (c) mRNA expression. Results are represented as mean ± SEM and normalized to fresh RPVs. *n* = 5 − 9. ^∗^*p* < 0.05 versus fresh. ^#^*p* < 0.05 versus unstretched.

Mechanical stretch downregulates the expression of APN in VSMCs (Figure 1), but whether it affects the expression of its receptors remains unclear. To elicit its intracellular effects, APN binds to its receptors AdipoR1, AdipoR2, and T-cadherin [39–41]. To investigate whether mechanical stretch affects the expression of these receptors, real-time PCR analysis was performed to study their mRNA expression levels in RPVs stretched for 6, 15, or 24 hours. As shown in Figure 2(a), AdipoR1 mRNA expression was not affected by mechanical stretch for 6 hours. Stretching the RPV for 15 hours, however, induced a significant increase in AdipoR1 mRNA expression as compared to fresh RPVs and unstretched RPVs for 15 hours (Figure 2(a)). Stretching the vessels for 24 hours also induced a significant upregulation in AdipoR1 mRNA expression compared to fresh RPVs (Figure 2(a)). These data indicate that mechanical stretch upregulates AdipoR1 gene transcription with a peak at 15 hours of stretch.

Stretching the RPVs for either 15 hours or 24 hours significantly upregulated AdipoR2 mRNA expression compared to fresh RPVs (Figure 2(b)), indicating that mechanical stretch also promotes an increase in AdipoR2 gene transcription.  Figure 2(c) shows that mechanically stretching the RPV for 6 hours slightly increased T-cadherin mRNA expression as compared to fresh and unstretched RPVs for 6 hours. In response to 15 hours of stretch, T-cadherin mRNA expression level increased significantly compared to fresh RPVs, while mechanical stretch for 24 hours did not significantly affect T-cadherin mRNA expression (Figure 2(c)). Thus, mechanical stretch upregulates T-cadherin gene expression after 15 hours in VSMCs. Collectively, these data indicate that mechanical stretch, which downregulates the expression of APN, induces an upregulation in the expression of the APN receptors, perhaps in an attempt to compensate for the reduced APN levels.

### 3.3. Mechanical Stretch Reduces the APN/Leptin Ratio in VSMCs

**Figure 3:**
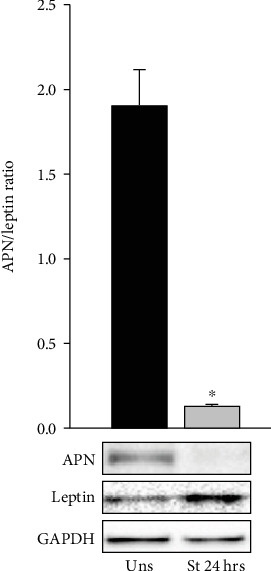
Mechanical stretch-induced reduction in the APN/leptin ratio in VSMCs. RPVs were mechanically stretched (St) for 24 hours or left unstretched (Uns), followed by Western blot analysis to study endogenous protein expression of APN and leptin in VSMCs. Results are represented as mean ± SEM. *n* = 4. ^∗^*p* < 0.05 versus unstretched.

The plasma leptin/APN ratio is emerging as a marker for metabolic syndrome and insulin resistance [65, 66]. To study the effect of mechanical stretch on the ratio of APN/leptin expression in VSMCs, RPVs were stretched for 24 hours followed by Western blot analysis to detect and measure endogenous APN and leptin levels.  Figure 3 reveals that the ratio of APN/leptin was significantly decreased by mechanical stretch for 24 hours, indicating that the hypertensive state is characterized by a low APN/leptin ratio not only in the plasma but also within VSMCs.

### 3.4. APN Attenuates Mechanical Stretch-Induced VSMC Hypertrophy

We have previously shown that both mechanical stretch and leptin increase VSMC hypertrophy [22, 23, 57]. On the other hand, APN has been reported as a cardioprotective protein that attenuates pressure overload-induced cardiac hypertrophy and protects against myocardial injury after ischemia-reperfusion [37, 38]. However, it is unclear whether APN exerts a vascular protective effect against hypertension-induced vascular hypertrophy. In order to examine this, RPVs were cultured mechanically stretched for 3 days or left unstretched with or without APN (10 *μ*g/ml), and changes in wet weight and protein synthesis (using [^3^H]-leucine incorporation) were measured [57]. 10 *μ*g/ml of APN was used because this concentration belongs to the physiological range of APN, which is 5 to 25 *μ*g/ml [62].

**Figure 4:**
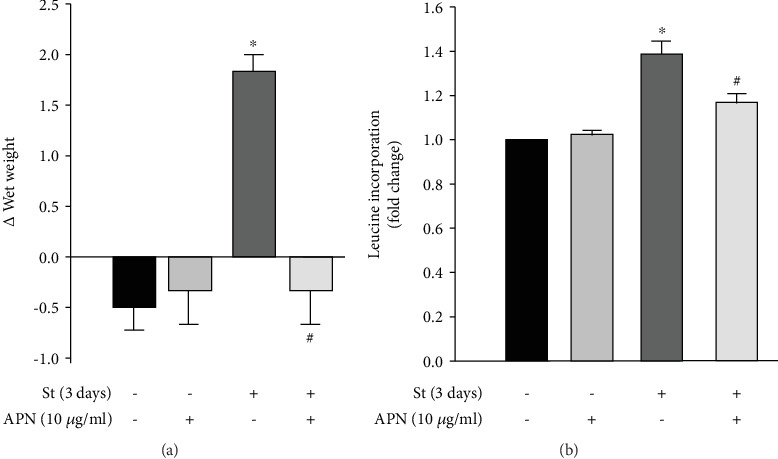
Exogenous APN-induced attenuation of mechanical stretch-mediated VSMC hypertrophy. RPVs were mechanically stretched (St) for 3 days or left unstretched with or without APN (10 *μ*g/ml). Changes in wet weight (a) and [^3^H]-leucine incorporation (b) were measured as indicators for hypertrophy. Results are represented as mean ± SEM. *n* = 6. ^∗^*p* < 0.05 versus unstretched. ^#^*p* < 0.05 versus stretched.

As shown in Figures 4(a) and 4(b), mechanically stretching RPVs for 3 days significantly increased their wet weight and protein synthesis compared to unstretched RPVs. Treating unstretched RPVs with exogenous APN (10 *μ*g/ml) had no effect on wet weight change (Figure 4(a)) or protein synthesis (Figure 4(b)), whereas treating mechanically stretched RPVs with APN significantly attenuated the stretch-induced increase in wet weight (Figure 4(a)) and protein synthesis (Figure 4(b)). These results indicate that APN exerts a protective effect on the vasculature under mechanical stretch by inducing an antihypertrophic effect on VSMCs. The ratio of dry weight to wet weight was also assessed in order to examine the possibility of water retention or osmosis as a reason for hypertrophy. The different groups did not have significant changes in dry weight/wet weight ratios, indicating that hypertrophy is not due to osmosis, but rather to protein synthesis.

### 3.5. APN Increases LKB1 and AMPK Phosphorylation in Mechanically Stretched RPVs

**Figure 5:**
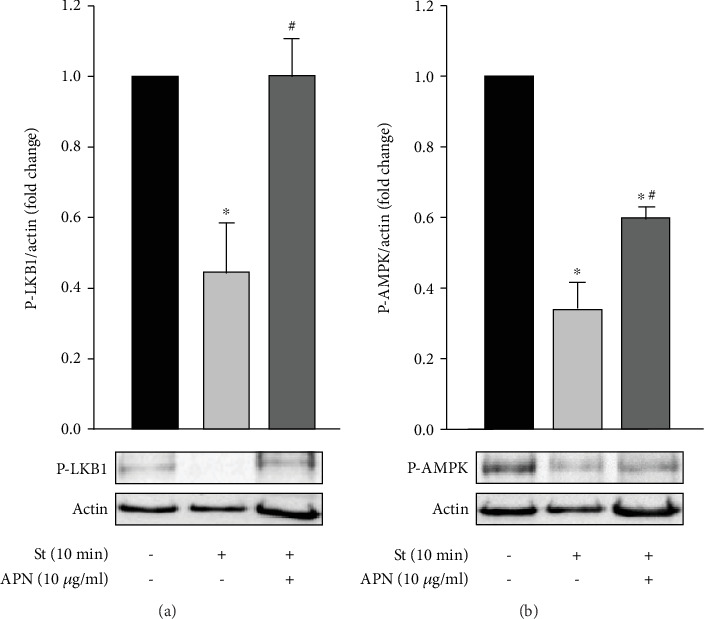
Exogenous APN-mediated increase in mechanical stretch-induced LKB1 and AMPK phosphorylation in VSMCs. RPVs were stretched for 10 minutes (St) and treated with APN (10 *μ*g/ml), followed by Western blot analysis to study LKB1 (a) and AMPK (b) phosphorylation. Data are represented as mean ± SEM and normalized to the unstretched RPVs. *n* = 4 − 7. ^∗^*p* < 0.05 versus unstretched. ^#^*p* < 0.05 versus stretched.

AMPK and its upstream kinase LKB1 exert protective cellular effects in diabetes and are activated by diabetic treatments like metformin [47, 51]. Moreover, AMPK activation has been shown to attenuate VSMC contractility, reduce blood pressure, and decrease VSMC hypertrophy [50, 52]. To study the effect of mechanical stretch on AMPK and LKB1 activation in VSMCs, RPVs were stretched for 10 minutes followed by Western blot analysis. As shown in Figures 5(a) and 5(b), mechanical stretch significantly reduced both LKB1 and AMPK phosphorylation after 10 minutes, indicating that the detrimental effects of stretch on the vasculature are likely to be mediated by reduced activation of LKB1 and AMPK and thus an attenuation of their protective effects.

To examine whether APN's observed antihypertrophic effect on VSMCs in response to mechanical stretch is mediated by LKB1-AMPK signaling, RPVs were treated with APN (10 *μ*g/ml) and stretched for 10 minutes. Western blot analysis was then performed to assess LKB1 and AMPK phosphorylation. Exogenous APN significantly increased LKB1 and AMPK phosphorylation in mechanically stretched RPVs compared to the untreated, stretched RPVs (Figures 5(a) and 5(b)). Thus, APN exerts a protective effect on VSMCs by activating LKB1-AMPK signaling under mechanical stretch. It is important to note that AMPK phosphorylation, although significantly increased by APN in stretched RPVs, remained significantly lower than unstretched RPVs, indicating that perhaps other signaling pathways activated by mechanical stretch are causing AMPK dephosphorylation.

### 3.6. APN Increases eNOS Activation in Mechanically Stretched RPVs

**Figure 6:**
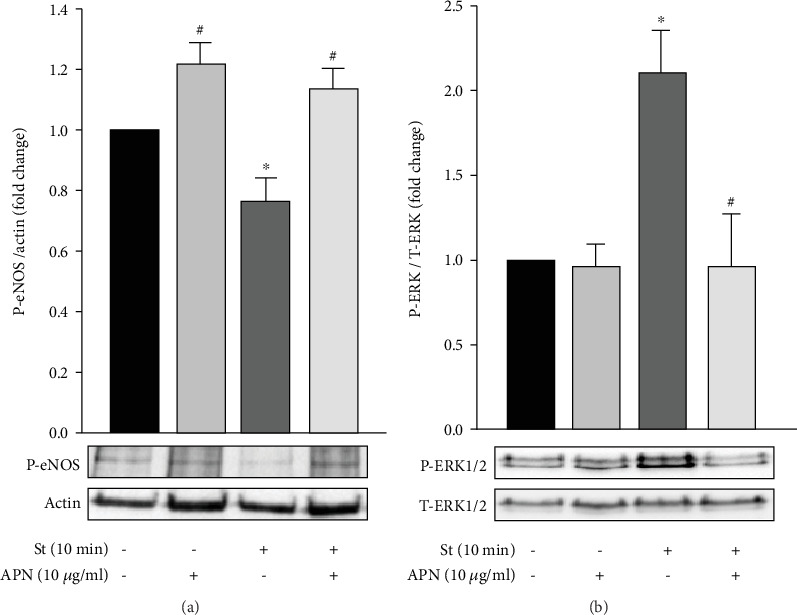
APN-mediated changes in mechanical stretch-induced eNOS and ERK1/2 phosphorylation in VSMCs. RPVs were stretched for 10 minutes (St) or left unstretched and treated with exogenous APN (10 *μ*g/ml), followed by Western blot analysis to detect eNOS phosphorylation (a) and ERK1/2 phosphorylation (b). Results are represented as mean ± SEM and normalized to the unstretched RPVs. *n* = 4 − 12. ^∗^*p* < 0.05 versus unstretched. ^#^*p* < 0.05 versus stretched.

When activated by phosphorylation at the Ser1177 residue, eNOS exerts a protective role on the vasculature by producing NO, which promotes vasorelaxation and exerts antihypertrophic effects on VSMCs [67, 68]. However, hypertension is associated with both hypertrophy and an impaired vasorelaxation response [23, 69, 70]. To examine the effect of mechanical stretch on eNOS phosphorylation at Ser1177 (which marks its activation), RPVs were stretched for 10 minutes followed by Western blot using a specific antibody that recognizes the phosphate group at Ser1177. Mechanically stretching the RPVs for 10 minutes significantly decreased eNOS phosphorylation compared to unstretched RPVs (Figure 6(a)), suggesting that the harmful effects of stretch are mediated by reduced eNOS activation.

To examine whether eNOS is involved in APN's observed antihypertrophic effect on mechanical stretch-induced VSMC hypertrophy, RPVs were treated with exogenous APN (10 *μ*g/ml) and either stretched for 10 minutes or left unstretched. As shown in Figure 6(a), APN increased eNOS activation in mechanically stretched vessels compared to untreated stretched RPVs. APN treatment on unstretched RPVs also significantly increased eNOS phosphorylation compared to stretched RPVs. These findings suggest that APN's protective effect against stretch-induced VSMC remodeling is mediated by activating eNOS.

### 3.7. APN Inhibits Mechanical Stretch-Induced ERK1/2 Phosphorylation in VSMCs

One of the mechanisms by which mechanical stretch leads to VSMC hypertrophy is by inducing ERK1/2 phosphorylation and subsequent activation [23, 56, 57, 60]. To investigate whether APN induces its antihypertrophic effect on mechanically stretched RPVs by affecting ERK1/2 signaling, RPVs were treated with exogenous APN (10 *μ*g/ml) and stretched for 10 minutes, followed by Western blot analysis to examine ERK1/2 phosphorylation. Mechanically stretching the RPVs for 10 minutes significantly increased ERK1/2 phosphorylation compared to unstretched RPVs (Figure 6(b)). Treating unstretched RPVs with exogenous APN (10 *μ*g/ml) had no effect on ERK1/2 activation (Figure 6(b)). Treating stretched RPVs with 10 *μ*g/ml of APN significantly reduced mechanical stretch-induced ERK1/2 phosphorylation (Figure 6(b)). Therefore, one of the mechanisms by which APN exerts its antihypertrophic effect on mechanical stretch-induced VSMC hypertrophy is by inhibiting ERK1/2.

### 3.8. APN Attenuates Mechanical Stretch-Induced ROS Formation in VSMCs

**Figure 7:**
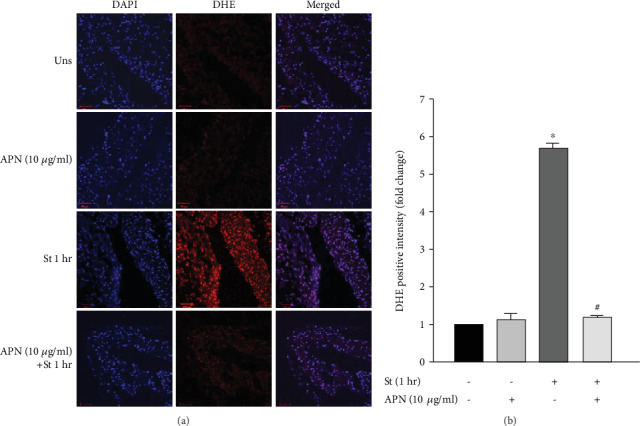
Exogenous APN-induced reduction in mechanical stretch-mediated ROS production in VSMCs. RPVs were unstretched (Uns) or stretched (St) for 1 hour and treated with 10 *μ*g/ml of APN. (a) Cryosections of the RPV wall were stained with DHE to detect ROS (red), and DAPI was used to detect the nuclei (blue) *(40x)*. (b) DHE fluorescence intensity was measured using ZEN software. Data are represented as mean ± SEM and normalized to the unstretched RPVs. *n* = 4. ^∗^*p* < 0.05 versus unstretched. ^#^*p* < 0.05 versus stretched.

We have previously studied the effect of mechanical stretch, mimicking hypertension, on ROS production in VSMCs [22]. Mechanical stretch increases ROS, which in turn promote vascular remodeling and induce hypertrophy [29, 30]. To investigate the effect of APN on stretch-induced ROS production in VSMCs, RPVs were stretched for 1 hour and treated with APN (10 *μ*g/ml), and DHE was used to detect ROS. As shown in Figure 7, unstretched RPVs that were treated with APN (10 *μ*g/ml) did not exhibit any marked changes in ROS production. Mechanical stretch significantly increased ROS production after 1 hour, while treating stretched RPVs with 10 *μ*g/ml of APN significantly decreased ROS generation (Figures 7(a) and 7(b)), indicating that APN's antihypertrophic effect on VSMCs during mechanical stretch is likely mediated by a reduction in ROS production.

### 3.9. APN Attenuates Leptin-Induced GATA-4 Nuclear Translocation in VSMCs

**Figure 8:**
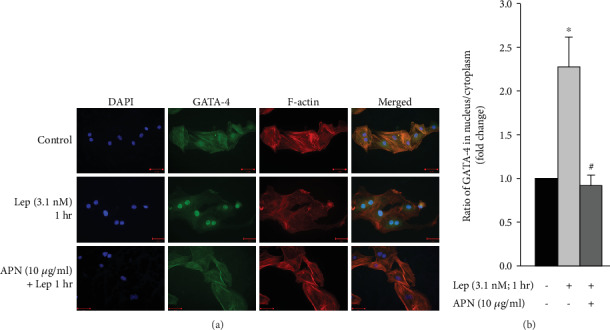
APN-mediated attenuation of leptin-induced GATA-4 nuclear translocation. RASMCs were treated with leptin (Lep; 3.1 nM) for 1 hour with or without APN (10 *μ*g/ml). (a) GATA-4 was visualized using anti-GATA-4 primary antibody and secondary antibody conjugated to CruzFluor 488 (green; second panel). DAPI was used to stain the nuclei blue (first panel), while Acti-stain 555 phalloidin stained F-actin (red; third panel). The overlay of DAPI, GATA-4, and F-actin is shown in the right panel (merged) *(40x)*. (b) GATA-4 fluorescence intensity was measured using ZEN software, and the ratio of GATA-4 in the nucleus/cytoplasm was assessed. Results are represented as mean ± SEM. *n* = 4. ^∗^*p* < 0.05 versus untreated. ^#^*p* < 0.05 versus leptin treatment.

Exogenous leptin activates translocation of the prohypertrophic transcription factor GATA-4 from the cytoplasm to the nucleus in RASMCs [22], indicating that leptin-induced GATA-4 nuclear translocation is likely a prominent mechanism by which leptin induces VSMC hypertrophy. To study whether GATA-4 is involved in the pathway of APN-induced attenuation of VSMC hypertrophy, RASMCs were pretreated with APN (10 *μ*g/ml), followed by leptin (3.1 nM) addition for 1 hour. Immunofluorescence was then performed to detect GATA-4 by using anti-GATA-4 antibody and secondary antibody conjugated to CruzFluor 488. As shown in Figure 8, exogenous leptin for 1 hour markedly increased GATA-4 nuclear translocation, whereas APN significantly attenuated leptin-induced GATA-4 nuclear translocation. These findings indicate that APN most likely inhibits VSMC hypertrophy by attenuating GATA-4 activation.

## 4. Discussion

The major findings in this study are as follows: (1) VSMCs synthesize APN and express its receptors. (2) Mechanical stretch, which mimics hypertension, decreases APN expression and increases leptin synthesis in VSMCs, thereby decreasing the APN/leptin ratio in VSMCs. (3) APN exerts an antihypertrophic effect on mechanical stretch-induced VSMC hypertrophy. (4) APN attenuates mechanical stretch-induced vascular remodeling by inhibiting ERK1/2 phosphorylation and ROS production and by increasing LKB1, AMPK, and eNOS phosphorylation. (5) APN attenuates leptin-induced GATA-4 nuclear translocation in VSMCs.

The force exerted by blood pressure on the vascular wall continuously exposes it to mechanical stretch. The higher the blood pressure, the higher the force of stretch, leading to vascular remodeling and hypertrophy [23, 70, 71]. Although it is a compensatory mechanism to hypertension, vascular hypertrophy is detrimental because it structurally and functionally changes the blood vessels, leading to reduced tissue perfusion and further inducing hypertension [72]. In order to study mechanical stretch-induced vascular hypertrophy, RPV organ culture was used as a well-established model to mimic hypertension [23, 25, 56, 57]. Being the bulk of the RPV wall, the pronounced longitudinal muscular coat makes it an ideal vessel to stretch by weight loading at a force that leads to 10-15% stretch, which has been calculated using the force-length relationship [57, 58, 60]. Mechanical stretch preserves the differentiated, contractile phenotype of the VSMCs in this *ex vivo* model of organ culture, as evidenced by the increased force of contraction and preservation of SM22 expression, a differentiation marker of VSMCs [56, 57, 73]. Moreover, this pre- and postcapillary blood vessel has spontaneous myogenic activity and has been used as an analogue for small precapillary resistance blood vessels [57, 58, 61].

Upon their discovery and for some time, leptin and APN were believed to be almost exclusively produced by adipocytes [9–15, 74]. We have shown that VSMCs produce the leptin protein and that its production is significantly upregulated by mimicking hypertension [22]. However, little is known about whether VSMCs synthesize APN and whether hypertension affects its production at the site of VSMCs. We began our research by investigating these two questions and found that APN is indeed produced by VSMCs and that the VSMC synthesis of APN is decreased by mimicking hypertension (Figure 1). When RPVs were stretched for 24 hours, APN expression in the VSMCs was significantly downregulated (Figure 1). Real-time PCR analysis was also performed to examine the effect of 6, 15, and 24 hours of mechanical stretch on APN mRNA expression and showed that mechanical stretch decreased APN mRNA expression in a time-dependent manner (Figure 1(d)). Thus, APN is indeed expressed in VSMCs, not only in adipocytes, and mechanical stretch downregulates its expression at both the gene and protein expression levels. Using real-time PCR analysis, we also examined the mRNA expression of the APN receptors AdipoR1, AdipoR2, and T-cadherin and found that they are all expressed in VSMCs. Mechanical stretch increased the mRNA expression of these receptors, perhaps as a feedback mechanism to compensate for the reduced levels of APN in the VSMCs as well as in the circulation during hypertension (Figure 2).

Although our findings are consistent with the prior knowledge that circulating plasma APN levels are decreased [6, 8] and leptin levels are increased [7] in hypertensive patients, their dysregulated synthesis and expression by VSMCs in hypertension proposes a new mechanism and possible explanation for this process, as opposed to just their production by adipocytes. The APN/leptin ratio in VSMCs was drastically reduced by mechanical stretch compared to unstretched vessels (Figure 3). The ratio of circulating APN/leptin is emerging as a marker for metabolic syndrome [65, 66], which includes hypertension, and our results indicate that VSMCs can now be viewed as contributors to this change in ratio.

Adiponectin supplementation has been shown to inhibit pressure overload-induced cardiac hypertrophy and protect against myocardial injury after ischemia-reperfusion [37, 38]. Zeidan et al. have previously shown that mechanical stretch and leptin, individually and together, significantly induce VSMC hypertrophy [23]. However, whether APN exerts a vascular protective or harmful effect on hypertension-induced VSMC hypertrophy has not been fully elucidated yet. In order to examine this, we treated RPVs with 10 *μ*g/ml of APN, which belongs to its normal physiological range [62].

The adiponectin used in our experiments is the purified recombinant murine globular domain of adiponectin. Being the larger portion of full-length adiponectin, this globular domain has been shown to exhibit greater potency than full-length adiponectin [75–79], which is why we decided to conduct our study using this form. However, the next step of our experimental investigation will focus on using the metabolically active high molecular weight oligomer and compare its effect with the low molecular weight trimer and the medium molecular weight hexamer in order to decipher which form of adiponectin is most potent in attenuating hypertension-induced vascular remodeling.

Hypertrophy was evaluated by the hypertrophic markers wet weight change and [^3^H]-leucine incorporation. APN significantly attenuated mechanical stretch-induced RPV hypertrophy by decreasing both weight change and protein synthesis in stretched vessels (Figure 4). Thus, APN exerts a protective, antihypertrophic effect against mechanical stretch-induced VSMC hypertrophy.

APN activates AMPK in several cell types, including ECs, VSMCs, and skeletal muscle cells [80, 81]. AMPK has been shown to exert several protective effects, such as attenuating VSMC hypertrophy [50], reducing blood pressure [52], and improving endothelial function [51]. We were interested in studying whether AMPK and its upstream kinase LKB1 were involved in the mechanotransduction of mechanical stretch-induced vascular remodeling. RPVs were stretched for 10 minutes, which corresponds to a time-point of significant LKB1 and AMPK phosphorylation in VSMCs [50]. To detect activation of LKB1 and AMPK, specific antibodies were used that mark their activating phosphorylation sites at Ser428 and Thr172, respectively. Mechanically stretching the RPV significantly reduced LKB1 and AMPK phosphorylation (Figure 5), adding LKB1-AMPK signaling to the pathways involved in stretch-induced VSMC remodeling.

When RPVs were treated with APN (10 *μ*g/ml) and mechanically stretched, LKB1 and AMPK phosphorylation was rescued (Figure 5), pointing towards a potential mechanism by which this protective hormone attenuates mechanical stretch-induced VSMC hypertrophy. In the APN-treated stretched vessels, LKB1 phosphorylation increased back to control (untreated and unstretched) levels, but AMPK phosphorylation, although significantly higher than that in untreated stretched RPVs, remained significantly lower than control levels (Figure 5). This may be due to other signaling pathways that are activated by mechanical stretch that APN does not attenuate. Future studies will investigate this notion. Moreover, it is particularly interesting to see that APN increased LKB1 activation in VSMCs, because, to our knowledge, the effect of APN on LKB1 activation has not been studied in VSMCs.

Downstream to AMPK activation is the phosphorylation and subsequent activation of the enzyme eNOS in ECs and cardiomyocytes [82–84]. When eNOS is activated by phosphorylation at its Ser1177 residue [85, 86], it produces NO which exerts protective actions on the vasculature that include antihypertrophic effects in VSMCs [63, 67, 68]. Moreover, APN has been shown to attenuate angiotensin II-induced contractility in a NO-dependent manner [63]. Although it was thought that VSMCs lack eNOS, studies have shown that eNOS is also expressed in VSMCs [87, 88], and our data has shown that mechanical stretch reduces eNOS phosphorylation at Ser1177 (Figure 6(a)). This is consistent with the knowledge that hypertension is associated with an impaired vasorelaxation response [69]. Moreover, when RPVs were treated with APN (10 *μ*g/ml), eNOS phosphorylation increased in both unstretched and stretched RPVs (Figure 6(a)), indicating that APN's protective antihypertrophic effect most likely occurs via the LKB1-AMPK-eNOS signaling axis in VSMCs.

NO has been shown to exert protective effects against vascular hypertrophy by inhibiting ERK1/2 activation in VSMCs [68], and research has shown that activated ERK1/2 itself mediates mechanical stretch-induced VSMC hypertrophy [23, 56, 57, 60]. In our study, mechanical stretch for 10 minutes (a time point of significant ERK1/2 phosphorylation [23, 29]) significantly increased ERK1/2 phosphorylation (Figure 6(b)), indicating a mechanism by which mechanical stretch induces VSMC hypertrophy. When stretched RPVs were treated with 5 *μ*g/ml of APN, ERK1/2 phosphorylation decreased, but this was not statistically significant (data not shown). When the concentration of APN was increased to 10 *μ*g/ml, which still belongs to the lower range of normal physiological APN concentration, ERK1/2 phosphorylation was significantly reduced in mechanically stretched RPVs compared to untreated stretched RPVs (Figure 6(b)). Thus, APN exerts its antihypertrophic effects on VSMCs by reducing ERK1/2 activation.

Mechanical stretch-induced vascular remodeling has been associated with increased ROS production in the vascular wall [22]. Vessels exposed to hypertension produce excessive levels of ROS [28], which in turn promote vascular hypertrophy [29, 30]. To investigate whether ROS production belongs to the mechanism of APN-induced attenuation of VSMC remodeling, RPVs were treated with APN and mechanically stretched for 1 hour, a time-point of significant ROS production in response to stretch [22]. Treatment with 5 *μ*g/ml of APN significantly reduced mechanical stretch-induced ROS formation (data not shown), while 10 *μ*g/ml of APN even further reduced ROS generation (Figure 7). Thus, APN exerts a protective effect on VSMC remodeling via reduction of mechanical stretch-induced ROS production, indicating the potential for APN as an anti-oxidant in the vasculature during hypertension. Interestingly, mechanical stretch increases the expression of leptin protein, which has a pro-oxidative effect [22] but decreases that of APN, which has an anti-oxidative effect. Whether the observed increase in ROS production in the RPV was directly induced by the upregulation of leptin and downregulation of APN has not been elucidated yet. Future studies will aim at examining this.

GATA-4 is a transcription factor that promotes cardiac hypertrophy by translocating to the nucleus and activating hypertrophic gene expression [26, 89, 90]. Research by Zeidan et al. has shown that exogenous leptin (3.1 nM) activates GATA-4 in cardiomyocytes [91], a mechanism by which leptin induces cardiomyocyte hypertrophy. In VSMCs, exogenous leptin (3.1 nM), which also induces VSMC hypertrophy [22, 23], activates GATA-4 nuclear translocation in a time-dependent manner and markedly after 1 hour [22]. In this study, we investigated whether GATA-4 is involved in the molecular mechanisms of APN-induced attenuation of VSMC hypertrophy and found that exogenous APN (10 *μ*g/ml) significantly reduced leptin-induced GATA-4 nuclear translocation in RASMCs (Figure 8). Thus, the mechanism of leptin-induced vascular hypertrophy via GATA-4 nuclear translocation is inhibited by APN in VSMCs.

## 5. Conclusion

Our study identifies molecular mechanisms involved in mechanical stretch-induced vascular remodeling and the role of APN and leptin in this process. It also provides evidence of APN's important protective effect against VSMC remodeling during hypertension. APN attenuates hypertrophy, ERK1/2 phosphorylation, ROS production, and GATA-4 nuclear translocation in VSMCs. It also increases the activation of the protective enzymes LKB1, AMPK, and eNOS in mechanically stretched RPVs. Hence, APN supplementation, upregulating its endogenous production, or using an agonist that mimics its effects provide a promising potential therapeutic strategy in attenuating the detrimental vascular effects of hypertension.

## Data Availability

The data used to support the findings of this study are available from the corresponding author upon request.
